# Proceedings: Effect of lucanthone on the radiosensitivity of a mouse tumour.

**DOI:** 10.1038/bjc.1975.310

**Published:** 1975-12

**Authors:** K. R. Rao, H. Fritz-Niggli


### EFFECT OF LUCANTHONE ON THE RADIOSENSITIVITY OF A MOUSE TUMOUR.

K. R. RAO and H. FRITZ-NIGGLI, Radiobiology Institute of Zürich University.

Lucanthone is reported to be carcinostatic (Hirschberg *et al.*, *J. natn. Cancer Inst.*, 1959, **22**, 567) and a radiosensitizer (Bases, *Cancer Res.*, 1970, **30**, 2007). But in our study lucanthone alone (70 mg/kg body weight) had no lasting effect on the Ehrlich carcinoma in mice. Based on mitotic studies, 4-day old ascites tumour was not sensitized to x-rays when pretreated with lucanthone. Tumour growth, evaluated as the average weight of solid tumours or the number of tumour cells in ascites bearing mice, was not significantly different between the group treated with x-rays only and the one treated with lucanthone plus x-rays. Thus, lucanthone seems to enhance the radiosensitivity of normal cell systems (Michel, *Experientia*, 1974, **30**, 1195) but not of the tumour tested.


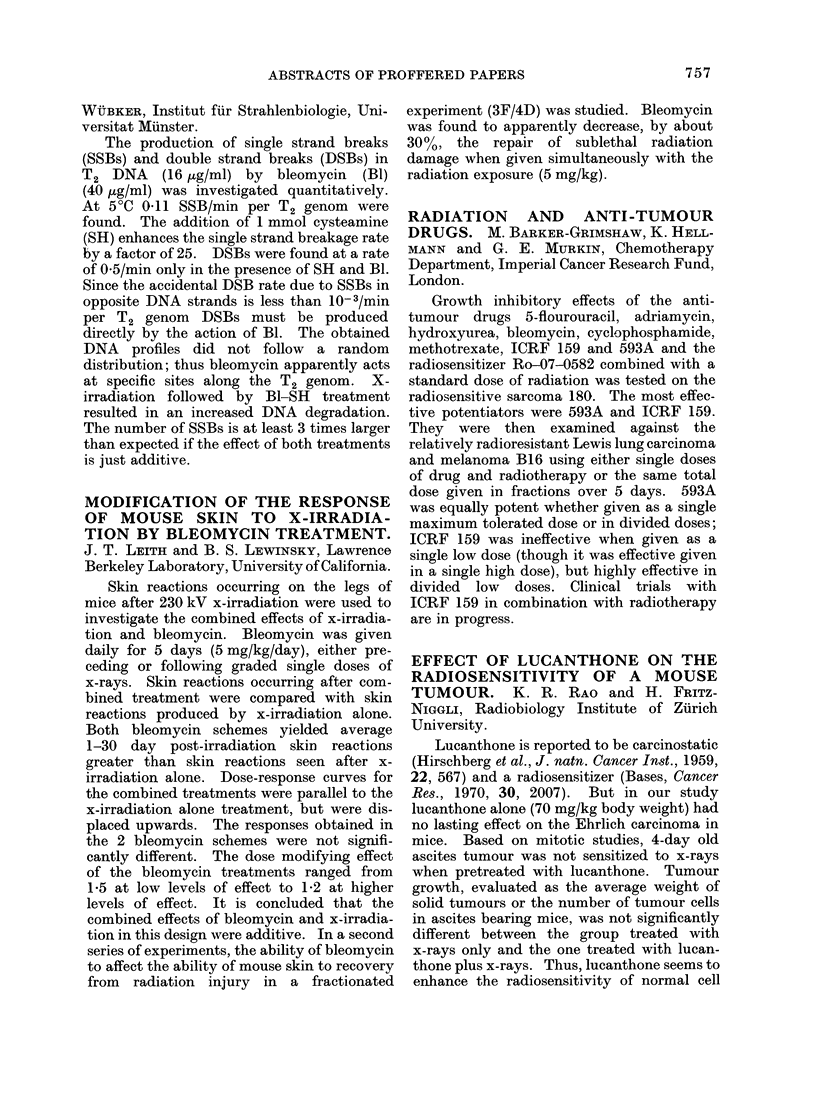

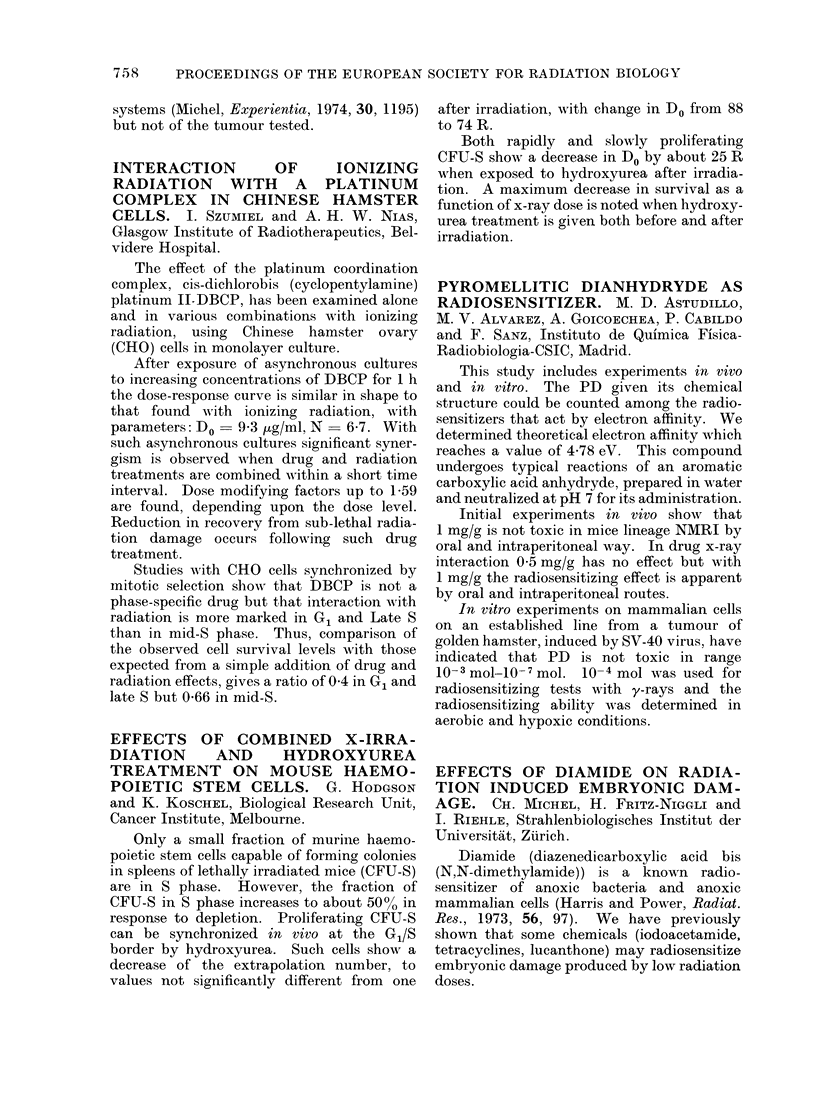


## References

[OCR_00011] Bases R. (1970). Enhancement of x-ray damage in HeLa cells by exposure to lucanthone (Miracil D) following radiation.. Cancer Res.

[OCR_00009] HIRSCHBERG E., GELLHORN A., MURRAY M. R., ELSLAGER E. F. (1959). Effects of miracil D, amodiaquin, and a series of other 10thiaxanthenones and 4-aminoquinolines against a variety of experimental tumors in vitro and in vivo.. J Natl Cancer Inst.

[OCR_00027] Michel C. (1974). Combined effects of Miracil-D and radiation on mouse embryos.. Experientia.

